# Use of a Smartphone Medication Reminder Application to Support Emerging Adult Adherence to Non-Antibiotic Treatment for Viral Upper Respiratory Tract Infection

**DOI:** 10.1177/21501319221129732

**Published:** 2022-10-13

**Authors:** David L. Brinker, Kasey A. Foley, Yanmengqian Zhou, Michelle Acevedo-Callejas, Yuwei Li, Erina L. Farrell

**Affiliations:** 1 Concord, MA, USA; 2 Center for Tobacco Products, U.S. Food and Drug Administration, Silver Spring, MD, USA; 3 The Pennsylvania State University, University Park, PA, USA

**Keywords:** antimicrobial stewardship, student health, emerging adults, medication reminder, smartphone app

## Abstract

**Objective:**

This research study is a test of the efficacy of a smartphone-installed medication reminder application to support provider-recommended treatment plans for young adult patients who were seen for upper respiratory tract infections (URTIs) and were not prescribed an antibiotic.

**Methods:**

Two hundred seventy-five patients seen at a university student health center for URTI symptoms were randomly assigned to the medication reminder app intervention or a control group and then surveyed both 1 and 14 days after their medical visits with questions about the treatment plan, their satisfaction with medical care, and the electronic support tools.

**Results:**

Compared to the control condition, patients using the reminder app reported more adherence to provider-recommended treatment plans. Patients with lower social support availability benefited more from being provided with these tools.

**Conclusion:**

These findings suggest that medication reminder apps have utility for increasing patient adherence to non-antibiotic URTI treatment plans, particularly among patients who lack high-quality informational and tangible social support.

**Innovation:**

This study demonstrates innovation in use of the medication reminder app to promote antibiotic stewardship with young adult patients in primary care.

## Introduction

Pharmaceutical antibiotics lose efficacy over time as their widespread usage accelerates selective pressures to favor the proliferation of resistant bacterial strands—a process referred to as “antibiotic resistance.” Despite the dire consequences of antibiotic resistance, unnecessary outpatient antibiotic prescribing remains very high in the U.S. Overprescribing is particularly common—exceeding 50%—for upper respiratory tract infections (URTIs),^1,2^ and often motivated by concerns unrelated to medical necessity, primarily that withholding antibiotics will reduce patient satisfaction, harm the provider-patient relationship, and motivate antibiotic-seeking elsewhere.^3-5^ Indeed, patients routinely expect antibiotics across a variety of common outpatient conditions,^6,7^ and those who do not receive antibiotics are often less satisfied with care than those who do.^8^

Evidence-based interventions are needed to improve patient satisfaction and treatment plan adherence when antibiotics are not prescribed. Post-visit support may help patients who are advised to utilize non-antibiotic treatment regimens to follow these recommendations, thereby relieving their symptoms until the underlying illness subsides, decreasing likelihood of antibiotic-seeking because they feel better, and increasing satisfaction with care over the course of the illness. One promising means of delivering this aid is through electronic patient supports. In this study, we examine the efficacy of a smartphone-installed medication reminder application on non-antibiotic treatment plan adherence for URTIs when utilized by young adult college students seen at a university student health center.

Medication reminder applications were initially developed for older adults and people with chronic disease who have multiple prescription medications, and they have shown considerable promise for improving patients’ condition management (eg, blood pressure monitoring), goal pursuit (eg, tracking weight loss), and medication adherence.^9,10^ Most traditional-age college students are emerging adults who are only gradually taking on more independence and responsibility for their own health and well-being, and may thus have limited experience caring for themselves during illness. In particular, many have previously relied on parents to help with choosing, obtaining, and administering both prescription and non-prescription drugs. With parents less involved, emerging adult patients may perceive a lack of support from health care providers who not only fail to prescribe the antibiotic they (mistakenly) expect, but also recommend what they perceive as a complex treatment regimen of over-the-counter medications and behavioral recommendations (eg, fluids, rest).

Feeling supported by health care providers is a significant predictor of adherence to medical treatment in multiple contexts.^11^ Patients who feel more capable of managing illness symptoms and confident of obtaining follow-up care if needed are also more positive toward non-antibiotic treatment.^12-14^ These findings suggest the value of interventions that scaffold patients’ efforts at appropriate and safe self-care while the illness runs its course. For emerging adult patients, use of a medication reminder app recommended by the provider may provide reassurance that the provider is caring and has chosen appropriate treatment, while also substituting for some of the practical assistance that parents would have given with treatment adherence. However, this intervention will likely have greatest impact on adherence for patients who were initially less willing to adhere to the non-antibiotic treatment. Thus, we hypothesize that:

*H1: Patient adherence to the treatment plan will be higher among users of a smartphone medication reminder application (compared to a non-user control group), and this effect will be stronger for patients whose initial adherence intention is weaker.*

Support from members of a patient’s social network predicts treatment adherence,^11,15^ and deficits in support hinder coping with illness.^16^ As emerging adults transition to taking responsibility for their own medical care, parents and guardians become less involved with their healthcare,^17^ to varying degrees.^18^ For emerging adult patients with lower support availability from their parents or other network members, additional support may be needed to promote treatment adherence. Indeed, additional sources of support are particularly beneficial for people with low network support availability.^19^ Because the medication reminder app we tested may provide supplemental support when a patient’s network support availability is low, we hypothesize that:

*H2: The effects of the smartphone medication reminder application will be strongest for patients with lower social support availability.*

## Methods

### Recruitment

Patients making appointments at the university’s student health clinic for URTIs were sent e-mails and text messages recruiting them to the study by clinic staff. Students who clicked on these messages were given more information about the study, and asked if they consented to installing the application on their personal smartphone and receiving text messages from the research team, and then directed to informed consent, a waiver of FERPA protection of student records relevant to the study, and a baseline questionnaire. Patients were excluded from the study if they had taken antibiotics in the week prior to the appointment, or if the diagnosis code in their post-visit electronic medical record was not consistent with URTIs.

### Data Collection and Measures

Upon arrival at their medical visits, patients were met by a research assistant who confirmed informed consent. Two hundred seventy-five patients were enrolled in the study. On completion of their medical visits, patients met with a research assistant again, who reviewed their prescription scripts, if any, to determine whether an antibiotic drug was prescribed. Patients were then assigned to experimental conditions. Patients who were prescribed antibiotics (n = 71) did not receive interventions. Patients who were not prescribed antibiotics (n = 204) were randomly assigned to treatment/control conditions to receive the smartphone application (n = 89) or to a control condition (n = 115). The analyses reported in this study pertain to patients who were not prescribed antibiotics.

Participants in the smartphone application condition installed the MediSafe^20^ smartphone application (www.medisafeapp.com) with help from a research assistant during the post-visit meeting. They loaded the treatment plan into the application, setting reminders as appropriate for each treatment. For example, a patient might enter Afrin, Tylenol, and fluids.

At the time of this experiment, some patients received text messages (These participants received regular text messages to their phones, which read “We hope your recovery is going well. If you need assistance from [clinic] healthcare providers, please call the 24/7 Advice Nurse at [phone number]. [Reply STOP to opt-out of reminders].”) from the research team with contact information for the clinic. Due to research assistant error, these text messages did not form a consistent experimental condition. Because these messages could also affect adherence, we tested the models in our analyses and determined that receipt of text messaging had no direct effect on adherence to treatment, nor did it moderate the effect of experimental condition on the outcomes we assessed.

Twenty-four hours after the medical visit, all patients received a survey invitation by email and were asked to complete it promptly. Patients who were not prescribed antibiotics received another survey 2 weeks later. Table 1 summarizes the means, standard deviations, and correlations of the variables used in this analysis.

**Table 1.** Variable Means, Standard Deviations, Correlations.

		Mean	s.d.	2	3	4	5	6	7
1	Treatment Certainty	4.49	0.72	.46***	.37***	.27**	.27*	.12	.20**
2	Treatment Understanding	4.24	0.95		.41***	.25**	.14	.18*	.26***
3	Adherence Intention	3.90	1.14			.14	.16	.21*	.60***
4	Information Support Quality	4.26	0.70				.45***	.43***	.18*
5	Tangible Support Quality	4.49	0.63					.33**	.08
6	Emotional Support Quality	4.55	0.60						.25**
7	Adherence	3.03	1.00						

* *P* < .05. **P < .01. ***P < .001.

The Day-1 survey instrument asked about the clinical visit and about the treatment plan that the provider recommended. Participants self-reported the treatment plan by entering each treatment recommendation into the survey. Patients were then asked to answer 3 questions assessing each treatment in the plan: *Treatment Certainty* (“how certain are you about how to follow this treatment [ie, what to do or how to do it]”), *Treatment Understanding* (“how well did you understand the provider’s reasons for recommending this treatment?”), and *Adherence Intention* (how likely are you to follow this treatment recommendation?). In the analyses, *Treatment Certainty and Treatment Understanding* are used as randomization checks, to ensure that patients assigned to different experimental conditions did not have significantly different assessments of their treatment plans. These ratings were given for each treatment recommendation, and we used the minimum values across treatments for each assessment in our analyses (ie, the lowest certainty, understanding, and intention values reported by the patient for any of their treatments). We did so because providers typically give several treatment recommendations within a plan, and while some may be easy to understand and comply with, others may be harder to understand and comply with. By using the minima, we identify patients who did not understand or did not plan to follow one or more aspects of the treatment plan.

The Day-14 survey instrument asked patients about those same treatments and for assessments of their medical care. Treatments from the Day-1 survey were automatically populated in the Day-14 survey, and patients reported *Adherence* with each one (“Thinking about any instructions you received from your provider, to what extent did you follow instructions for this treatment?”). We again used the minimum of these responses to individual treatments to measure the patient’s level of adherence to the treatment plan.

In the Day 14 survey, patients were also asked to report on anyone in their social networks (friends and family) who supported them during their illness. For each support person that the patient listed, we asked about 3 types of support they might have provided: informational (“Did this person give you informational support [information, suggestions, advice]?”), tangible (Did this person give you tangible support? [helping you physically or materially, such as by bringing you things or going places with you]?), and emotional (“Did this person provide emotional support [sympathy, compassion, caring, concern, being there]?”). For each of these types of support, we asked about the quality of that support: *Informational Support Quality* (“Please rate the quality of the informational support.”), *Emotional Support Quality* (“Please rate the quality of the emotional support.”), and *Tangible Support Quality* (“Please rate the quality of the tangible support.”). If a support person did not provide one of the types of support, we coded the quality as zero. We used the maximum quality of each support type received from any support person to assess the quality of each type of support available to the patient.

Finally, in the Day 14 survey, participants responded to items assessing the app’s usefulness and how long they continued to use it.

## Preliminary Analyses

### Randomization Check

We verified that randomization was successful by comparing the mean *Adherence Intention* of patients assigned to interventions and the patients assigned to control conditions. Assignment to the experimental condition did not predict *Adherence Intention* (*b* = −.04; *P* = .798). Assessments of *Treatment Certainty* and *Treatment Understanding* predicted *Adherence Intention* (*b* = .36; *P* = .002, *b* = .37; *P* < .001 respectively) and did not differ between experimental conditions (*b* = −.10; *P* = .336, *b* = .01; *P* *=* .941 respectively), Thus, the control and treatment groups did not differ in their Day 1 intentions to comply with treatment plans, and it can be assumed that later differences in adherence did not result from different initial intentions.

### Application Quality

Patients rated the application positively; 84% agreed that “this is a useful app,” and 55% agreed that “using this app improved my ability to manage symptoms with medications and other symptoms.” 75% of patients who installed the application used it until the illness subsided.

## Results

The analyses were conducted using IBM SPSS Statistics^21^ and R^22^ using the GLM and sjPlot^23^ packages. Because our hypotheses are directional, we assessed effects at a one-tailed alpha of .10. Of 204 enrolled participants who were assigned to the application or to the control condition, 196 completed the Day 14 survey (8 dropped out of the study or returned incomplete Day-14 surveys).

### Treatment Plan Adherence

**Figure 1.**
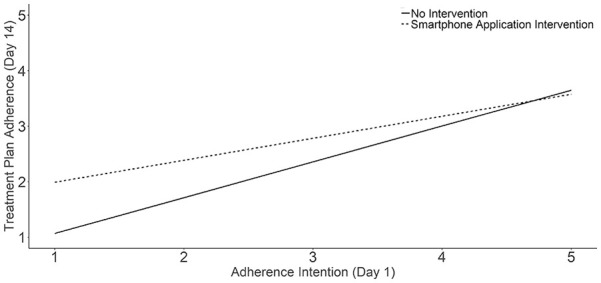
Smartphone application users had higher day-14 treatment plan adherence at lower levels of day-1 adherence intention.

We hypothesized that *Adherence* would be higher among subjects assigned to the smartphone application intervention. This hypothesis was not supported overall, but was supported for patients with lower *Adherence Intention.* Specifically, *Adherence* was higher among patients in the smartphone application condition who had lower *Compliance Intention*, compared to patients in the control condition with lower *Compliance Intention*. Figure 1 illustrates the mean differences. Statistical tests are reported in Table 2.

**Table 2.** Regression Results—Treatment Plan Adherence Regressed on Smartphone Application Intervention and Adherence Intention.

Predictors	DV: Treatment compliance		
	Estimates	CI	*P*
Intercept	0.42	−0.11 to 0.96	.121
Smartphone Application Group	1.17	0.39-1.96	.004
Adherence Intention	0.65	0.51-0.78	<.001
Smartphone Application Group × Adherence Intention	−0.25	−0.44 to −0.06	.013
Observations	196		
*R*^2^ Nagelkerke	.516		

### Social Support Availability

We hypothesized that the effects of the electronic patient support system would be stronger for patients with lower levels of social support availability. We modeled *Adherence* as a function of 3-way interactions between assignment to the smartphone application condition, *Adherence Intention*, and each of the 3 types of patient-rated social support quality.

Social support availability moderated the efficacy of the smartphone application on *Adherence*, particularly among participants with lower *Adherence Intention*. Table 3 shows the estimated marginal means for low *Adherence Intention* (1 *sd* below the mean) with and without the application and at high and low levels of each type of social support. Consistent with our hypothesis, the application condition had a larger association with Treatment Compliance among those with lower informational support and tangible support, compared to participants with higher levels of those support types. Contrary to our hypothesis, the application condition was associated with higher compliance among those with higher emotional support, but not for those with lower emotional support. Statistical tests are reported in Table 4.

**Table 3.** Estimated Marginal Means of Adherence for Low-Adherence-Intention Patients by Experimental Condition and Level of Social Support.

	Low support			High support		
	Control group	Treatment group	Delta (% increase)	Control group	Treatment group	Delta (% increase)
(A) Emotional Support	2.3	1.6	−30	2.1	3.4	62
(B) Informational Support	1.9	3.9	105	2.3	2.7	17
(C) Tangible Support	2.2	3.0	36	2.5	2.5	0

“High” and “Low” are ±1 *s.d.* from variable means. Cells are estimated marginal means (EMMs) for low levels of Adherence Intention at high and low values of each support type.

**Table 4.** Regression Results—Treatment Plan Adherence Regressed on Smartphone Application Intervention, Initial Adherence Intention, and Social Support Availability.

Predictors	DV: Treatment adherence		*P*
	Estimates	CI	
Intercept	0.09	−1.55 to 1.73	.915
Smartphone Application	0.19	−1.75 to 2.12	.851
Adherence Intention	0.68	0.29-1.08	.001
Informational Support Quality	0.27	−0.03 to 0.57	.084
Emotional Support Quality	−0.20	−0.66 to 0.26	.400
Tangible Support Quality	0.17	−0.10 to 0.44	.221
Smartphone Application × Adherence Intention	−0.00	−0.46 to 0.46	.996
Smartphone Application × Informational Support Quality	−1.07	−2.05 to −0.08	.035
Adherence Intention × Informational Support Quality	−0.06	−0.15 to 0.03	.166
Smartphone Application × Emotional Support Quality	1.50	0.45-2.55	.006
Adherence Intention × Emotional Support Quality	0.05	−0.06 to 0.17	.360
Smartphone Application × Tangible Support Quality	−0.43	−0.86 to −0.01	.046
Adherence Intention × Tangible Support Quality	−0.03	−0.10 to 0.03	.332
Smartphone Application × Adherence Intention × Informational Support Quality	0.25	−0.01 to 0.52	.064
Smartphone Application × Adherence Intention × Emotional Support Quality	−0.36	−0.63 to −0.08	.012
Smartphone Application × Adherence Intention × Tangible Support Quality	0.09	−0.01 to 0.20	.090
Observations		192	
*R*^2^ Nagelkerke		.593	

## Discussion and Conclusion

### Discussion

We conducted a randomized trial to test the efficacy of an electronic patient support administered following medical visits for URTIs not resulting in a prescription for antibiotics. Patient reminder systems vary in their features, such as the ability for patients to send messages back to providers, to upload patient data for provider review. The application in this study did not facilitate post-visit interaction between providers and patients, so it represents a “lower-tech” version of intervening with reminder applications. Although meta-analytic studies^24-26^ have noted differences in application features and deployment, it remains unclear which features are central to application effects on compliance. We recommend future research examine whether fuller-featured reminder applications have stronger effects in the context of symptom management for URTIs.

We found that patients with lower intention to adhere to treatment plans who were given a smartphone treatment reminder application reported adhering more diligently to the treatment plan compared to patients who did not receive the application. Patients who already intended to adhere to the treatment plan on Day 1 of the survey typically reported doing so on Day 14, and these patients were largely unaffected by use of the app. The intervention’s effect was more pronounced among those who lacked high-quality informational and tangible support in their social networks. The intervention also had a more pronounced effect among patients who reported having access to high-quality emotional support, but it was not more effective among those without it.

The findings of the study should be interpreted with its limitations in mind. This study was performed on a relatively small sample size; as with all intervention research, repeated trials are key to establishing the reliability of an effect. We also tested a single medication reminder application, and we did not require or monitor participants’ actual use of the app. Although participants evaluated Medisafe positively, findings might vary if other apps are evaluated more or less positively, and if apps are used to a greater or lesser extent. In our study, research assistants helped patients enter their treatment plans into the application. It is possible that the effect of the application on adherence was improved by this aspect of the intervention.

Our results are best generalized to the college student population. Young adults are likely to be especially comfortable using smartphone applications,^27^ and other populations may therefore respond less positively to the intervention we tested. Finally, we did not attempt to measure “notification fatigue,” which may decrease the positive influence of the application over time, perhaps especially if participants already receive many notifications from smartphone applications or have reminders set for longer durations.^28^

### Innovation

This study’s innovation is the use of a medication reminder app for a novel population and goal. Typically, medication reminder apps are recommended for use by the elderly, individuals with chronic illness, and those who have difficulty remembering their prescribed medications due to their quantity or complexity.^9,10^ Here, we employed the Medisafe app as a support for young adults with acute URTI, with the goal of improving adherence to non-antibiotic, largely over-the-counter treatment regimens. The findings indicate that this innovation has the potential to improve antibiotic stewardship in this group.

### Conclusion

Non-antibiotic treatment plans for URTIs are only as effective as patients’ willingness to adhere to them. These results suggest that clinics may find utility in adopting electronic patient supports to increase treatment plan adherence for URTIs, especially among patients who may have doubts or concerns about non-antibiotic treatment. In addition, patients lacking tangible and informational support may stand to benefit more, since the app can help to stand in for deficits in practical support from others. In practice, getting patients to use a medication reminder app is likely to require some additional visit time and instruction from providers or other staff, but the time and effort may pay dividends for non-antibiotic treatment adherence and reduced antibiotic-seeking.
